# A Comprehensive Review of the Antitumor Activity of Olive Compounds: The Case of Olive Oil, Pomace, and Leaf Extracts, Phenolic Alcohols, Secoiridoids, and Triterpenes

**DOI:** 10.3390/antiox14020237

**Published:** 2025-02-18

**Authors:** Diana Melo Ferreira, Maria Beatriz P. P. Oliveira, Rita Carneiro Alves

**Affiliations:** REQUIMTE/LAQV (Rede de Química e Tecnologia/Laboratório Associado para a Química Verde), Department of Chemical Sciences, Faculty of Pharmacy, University of Porto, Street of Jorge Viterbo Ferreira, 228, 4050-313 Porto, Portugalbeatoliv@ff.up.pt (M.B.P.P.O.)

**Keywords:** olive pomace, olive oil, olive leaf, phenolic compounds, hydroxytyrosol, oleuropein, oleocanthal, oleacein, maslinic acid, cancer

## Abstract

Olive oil is widely recognized for its cancer-prevention properties, and its by-products, such as pomace and leaves, offer an opportunity for compound extraction. This study comprehensively reviews the antitumor activities of olive extracts and compounds in both in vitro and in vivo studies. Key compounds, including hydroxytyrosol (HT), oleuropein (OL), oleocanthal (OC), and maslinic acid (MA), demonstrated significant antiproliferative, apoptotic, antimigratory, and anti-invasive effects, along with selective cytotoxicity, particularly against breast and colorectal cancer. HT, OC, and MA showed anti-angiogenic effects, while HT and OC showed antimetastatic effects. Moreover, HT, OL, and OC also presented synergistic effects when combined with anticancer drugs, improving their efficacy. Additionally, HT, OL, and MA exhibited protective effects against several side effects of chemotherapy. These compounds are able to modulate important signaling pathways such as the mammalian target of rapamycin, regulate oxidative stress through reactive oxygen species production, modulate angiogenic factors, and induce autophagy. Interestingly, the synergistic effects of the compounds within olive extracts appear to be stronger than their individual action. There is a need for dose optimization, further mechanistic studies to clarify the precise mechanisms of action, and future studies using olive pomace extracts with animal models.

## 1. Introduction

Cancer is a group of diseases characterized by uncontrolled abnormal cell proliferation, leading to tumor formation. It is the second leading cause of death globally, responsible for 9.6 million deaths in 2018, with lung, prostate, and colorectal cancers being the most common among men and breast, colorectal, and lung cancers being the most prevalent among women. Cancer imposes a substantial physical, emotional, and financial burden, especially in low- and middle-income countries where diagnosis and treatment options are limited [1].

Discovering new selective drugs is imperative as cancer incidence continues to escalate worldwide and current chemotherapeutic drugs cause significant toxicity on healthy tissues, with severe side effects such as nephrotoxicity (e.g., acute kidney injury), hepatotoxicity (e.g., steatosis), neurotoxicity (e.g., sensory neuropathy), and cardiotoxicity (e.g., heart failure) [2].

In addition, identifying natural compounds that can serve as anticancer agents may uncover potential therapeutic targets and minimize the side effects of chemotherapy. Plant polyphenols, known for their anti-aging, anti-inflammatory, antimicrobial, and antioxidant effects, target all phases of chemoprevention: (a) primary prevention by inhibiting cancer initiation, (b) secondary prevention by inhibiting cancer promotion, and (c) therapeutic intervention by inhibiting cancer progression [3,4].

The Mediterranean diet is widely recognized for its protective role against cancer due to the polyphenols found in olives (*Olea europaea* L.) and extra virgin olive oil (EVOO). Building on this premise, exploring the antitumor ability of potent natural extracts obtained from olive matrices such as olive by-products could benefit cancer prevention and treatment strategies [5,6,7]. This perspective becomes even more relevant if we consider that olive oil production generates thousands of tons of by-products which pose environmental concerns. In the 2023/24 harvest season, global olive oil production was approximately 2,564,000 tons. Within the European Union, Portugal’s production rose by 28%, totalizing 160,900 tons [8].

Olive pomace (OP) is the largest example of olive by-products. The ratio of OP to olive oil typically varies between 3 and 5 tons of OP. Consequently, in 2023/24, its global production is estimated to be between 7,692,000 and 12,820,000 tons [8]. Although OP is phytotoxic due to the limited transfer of polyphenols to olive oil, these compounds, if properly extracted, can serve as active ingredients in the development of functional foods, nutraceuticals, or anticancer drug formulations [9]. Similarly, olive leaves can offer additional value due to their promising pharmacological properties [10].

Olive leaves primarily result from tree pruning and olive harvesting, constituting approximately 10% of the total weight of harvested olives [11]. Assuming an average oil yield of 20% from olives, this production would require about 12,820,000 tons of olives. Consequently, olive leaves would have a weight of approximately 1,282,000 tons worldwide [8,12], depending on olive variety, cultivation practices, and regional differences [10,11].

Therefore, combining cancer treatment with olive compounds and extracts may offer synergistic protection against cancer progression due to their bioactivities [3,4]. For example, a functional ingredient obtained from OP demonstrated antimicrobial activity against *Staphylococcus aureus* and *Escherichia coli*, with the best minimal inhibitory concentration observed with higher hydroxytyrosol (HT) contents [13]. Other studies demonstrated the neuroprotective effects of olive extracts in preventing oxidative stress (OS) by reducing Aβ and tau protein aggregation in vivo [14,15].

This work aims to comprehensively review the studies available in the current literature regarding the antitumor activity of olive pomace extracts (OPEs). Due to the limited number of articles on OPE, studies involving olive oil extracts (OOEs) and olive leaf extracts (OLEs) were also included. Studies which used individual or multiple olive compounds were also included to provide a more complete overview, as these are the most available and extensively studied in the literature.

## 2. Methodology

The literature search was performed using PubMed, Scopus, ScienceDirect, Web of Science, and Google Scholar electronic databases and restricted to the years 2004 to 2024 to focus on data from the last 20 years. The search included the following keywords: “olive pomace”, “olive by-product”, “olive”, “olive oil”, “olive leaf”, “olive extract”, “phenolic compound”, “phenolic”, “polyphenol”, “tumor”, “antitumoral”, “antitumor”, “antitumorigenic”, “cancer”, “anticancer”, “anticarcinogenic”, “cytotoxicity”, “cytotoxic effect”, “apoptosis”, “apoptotic effect”, “antiproliferative effect”, “cancer cell line”, and “bioactivity”. Duplicated references were always deleted. The inclusion criteria were in vitro studies, which used cell lines, and in vivo studies, which used animal models. The exclusion criteria were human clinical trials. As a result of this search and criteria, 85 relevant articles were considered for elaboration for this review.

## 3. Antitumor Activity of Olive Compounds

**Figure 1:**
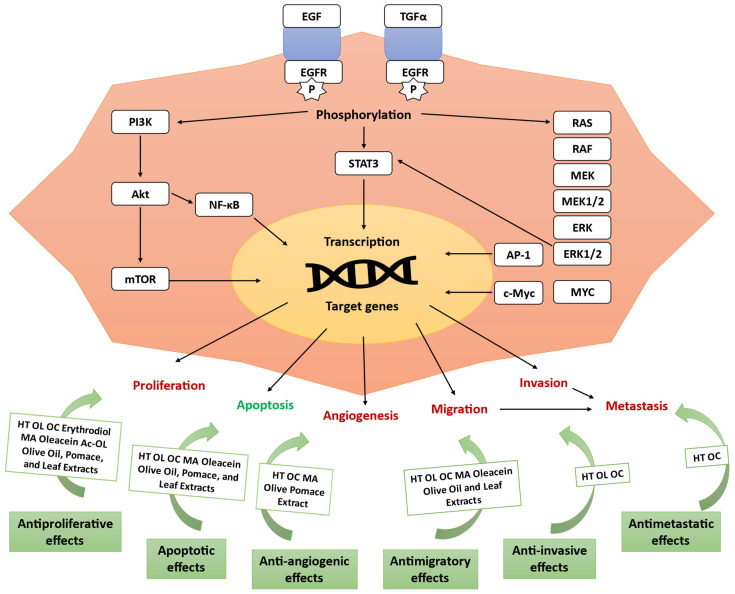
Cancer signaling pathways, adapted from Alam et al. (2022). Epidermal growth factor (EGF) and transforming growth factor (TGF) bind to EGF receptor (EGFR) in the cell membrane, triggering downstream cascades, leading to the activation of phosphatidylinositol-3-kinase (PI3K) and protein kinase B (Akt). The mammalian target of the rapamycin (mTOR) pathway regulates cell growth. Nuclear factor kappa-B (NF-κB) regulates inflammation, apoptosis, and cell cycle-related genes. Signal transducer and activator of transcription 3 (STAT3) drives the transcription of genes involved in cell survival, proliferation, and angiogenesis. EGFR also activates the RAS/RAF/MEK/ERK cascade, which leads to the activation of extracellular signal-regulated kinase 1/2 (ERK1/2) and the activating protein 1 (AP-1) [16]. Hydroxytyrosol (HT), oleuropein (OL), oleocanthal (OC), maslinic acid (MA), peracetylated oleuropein (Ac-OL).

Cancer develops due to aberrations in cellular signaling pathways, involving numerous molecular and key factors that drive uncontrolled growth [16]. Most studies discussed below have focused on these pathways, aiming to target them as potential cancer therapy. In fact, olive compounds have been reported to exert several antitumor effects (Figure 1).

**Figure 2:**
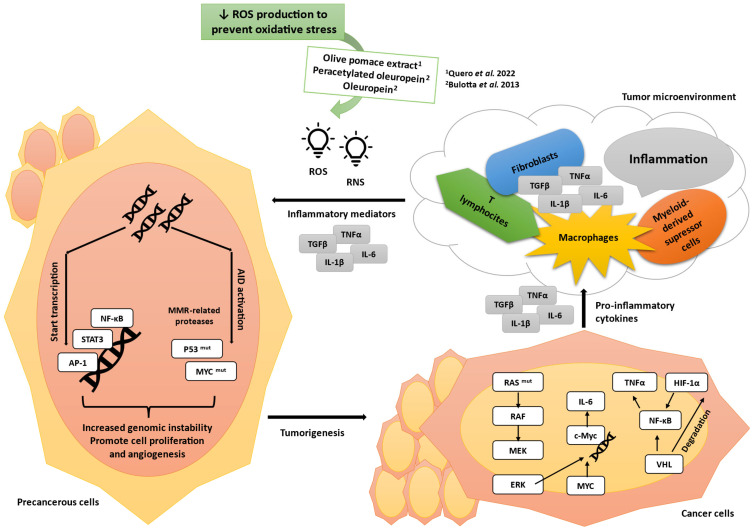
The role of inflammation in cancer, adapted from Wen et al. (2022). Inflammation triggers the release of inflammatory factors such as tumor necrosis factor (TNFα), transforming growth factor (TGFβ), and interleukins (ILs). Reactive oxygen species (ROS) and reactive nitrogen species (RNS), produced by macrophages, fibroblasts, and T lymphocytes, activate AID (activation-induced cytidine deaminase) and mutate P53 and MYC genes. Inflammation suppresses mismatch repair (MMR), leading to the accumulation of genetic alterations. Inflammation promotes the proliferation and survival of tumor progenitor cells by inducing the expression of growth factors and cytokines (NF-κB, STAT3, AP-1). RAS, MYC, and VHL genes can also promote tumor progression through the recruitment of pro-inflammatory cells to secrete inflammatory mediators, sustaining the inflammatory microenvironment [17]. A decrease in ROS production to prevent oxidative stress was observed in the studies of Quero et al. (2022) [4] and Bulotta et al. (2013) [19].

Inflammation (Figure 2) also contributes to tumor development as it induces angiogenesis [17]. Angiogenesis is tightly regulated by hypoxia inducible factor (HIF); vascular endothelial growth factor (VEGF); hepatocyte growth factor (HGF); matrix metalloproteinase (MMP); transforming growth factor (TGF); and tumor necrosis factor (TNF), which promote the proliferation of endothelial cells, leading to the formation of new blood vessels to provide nutrients and oxygen to cancer cells [18].

**Figure 3:**
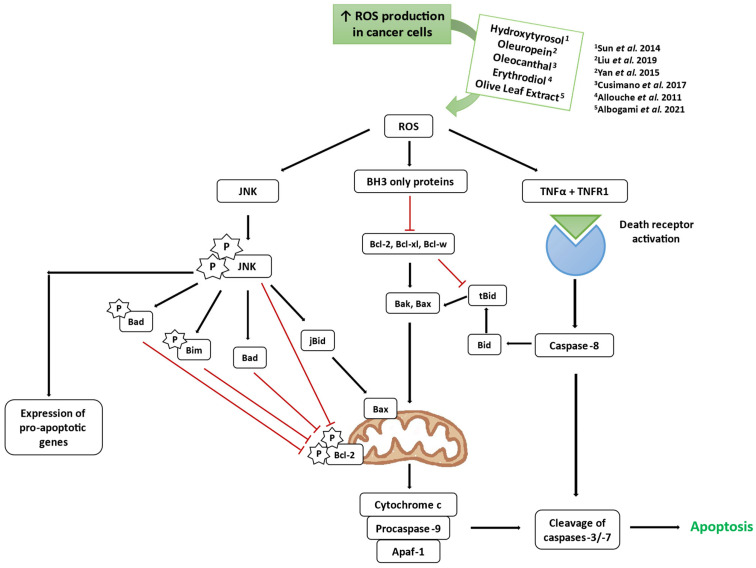
Role of reactive oxygen species (ROS) in apoptosis, adapted from Arfin et al. (2021). Mitochondria are a primary ROS source, regulated by nitric oxide (NO), Ca^2+^, and tumor necrosis factor (TNFα). Low ROS levels regulate hypoxia adaptation and pro-inflammatory cytokine production, while high ROS levels can be toxic and damage the mitochondrial membrane, releasing cytochrome c to the cytoplasm which induces caspases cleavage, resulting in apoptosis. TNFα ligand binds to TNFR1 death receptor, triggering the activation of caspase-8 and leading to the cleavage of caspase-3 [21]. An increase in ROS production in cancer cells was observed by Sun et al. (2014) [22], Liu et al. (2019) [23], Yan et al. (2015) [24], Cusimano et al. (2017) [25], Allouche et al. (2011) [26], and Albogami et al. (2021) [27].

Apoptosis (Figure 3), a programmed cell death mechanism marked by structural changes and DNA fragmentation, is often evaded by cancer cells [16]. It can occur via the intrinsic pathway, which involves mitochondrial release of pro-apoptotic factors, or the extrinsic pathway, triggered by death receptors [20].

Table 1 summarizes the antitumor activity of extracts from olive matrices. Olive oil, pomace, and leaf extracts are valuable products derived from the olive tree, each offering unique bioactive properties due to their richness in phenolic compounds [9,10,13].

Hydroxytyrosol (HT) and tyrosol (TYR) are key olive phenolic alcohols (Table 2). HT consists of a benzene ring with two hydroxyl groups (positions 3 and 4) and an ethanol side chain (position 2), while TYR lacks the hydroxyl group at position 3 [42].

Another important class is olive secoiridoids (Table 3), including oleuropein (OL), oleocanthal (OC), p-HPEA-EDA (dialdehydic form of decarboxymethyl ligstroside aglycone), oleacein, OL aglycone, and peracetylated oleuropein (Ac-OL). Structurally, these compounds are characterized by a secoiridoid skeleton, which includes a cyclopentane ring fused to a tetrahydrofuran ring. These molecules often possess hydroxyl and methoxy groups, which contribute to their potent antioxidant properties [56].

Additionally, olive pentacyclic triterpenes (Table 4) such as maslinic acid (MA), erythrodiol, oleanolic acid, and uvaol are characterized by a structure of five interconnected rings which form a rigid backbone. The structure allows for various functional groups to attach, leading to a diverse array of compounds with distinct biological activities [87].

Several compounds obtained from olive extracts exhibited strong antiproliferative, apoptotic, and cytotoxic effects against breast cancer models (Table 1). In particular, OL aglycone, isolated from EVOO, demonstrated superior efficacy in reducing cell viability compared to HT, TYR, and elenolic acid. Remarkably, the first compound also enhanced trastuzumab sensitivity in resistant cells [28].

In addition, polyphenols extracted from EVOO were as effective as lapatinib in suppressing FAS expression and inducing HER2 oncogene degradation. This is significant because it disrupts the lipogenic gain as FAS overexpression is linked to HER2 in breast cancer cells [30]. Similarly, OC combined with lapatinib synergistically inhibited the growth of HER2-positive cancer cells and suppressed the activation of HER2, EGFR, and c-MET pathways [64]. Additionally, HT also showed the ability to downregulate FAS in liver cancer cells [54].

OPEs also showed strong antiproliferative activity against breast cancer cells [5,9]. For example, a dry olive mill residue water extract demonstrated stronger antiproliferative effects than an OP water extract. However, HT, the major compound in both extracts, exhibited the highest antiproliferative potential, even stronger than 5-fluorouracil, a cytostatic agent. Interestingly, combining 5-fluorouracil with dry olive mill residue extract or HT enhanced its effects, with HT + 5-fluorouracil showing the strongest inhibition, emphasizing that HT could be the major contributor of the extracts’ effects [5].

The previous results are supported by other studies using individual compounds: HT had synergistic effects with paclitaxel in breast cancer models. One important aspect of the study was the assessment of the impact on oxidative status. Interestingly, the combined treatment resulted in lower DNA damage in lymphocytes, increased plasma antioxidant capacity, and reduced protein carbonyls [45].

Additionally, HT with cetuximab enhanced apoptosis through autophagic pathways in colorectal cancer cells [50], whereas OL achieved a synergistic effect with doxorubicin, sharply reducing tumor volume in vivo by inducing apoptosis via the mitochondrial pathway in breast cancer [59]. Moreover, OC may also enhance sensitivity to tamoxifen [62], and an OL-enriched extract enhanced the cytotoxicity of dacarbazine and everolimus in melanoma cells, outperforming OL used alone [76].

Overall, all of these studies highlight the synergistic potential of combining several olive compounds with standard chemotherapy drugs.

Regarding leaf extracts, an OLE decreased the viability of several breast cancer cell lines in a dose-dependent manner [31]. Similarly, other OLEs demonstrated antiproliferative effects on cancer cell lines (MCF-7 and T-24) and on endothelial cells (BBCE). This was also verified for luteolin and its glucosides. Additionally, mixtures of these compounds, mimicking the proportions found in the extracts, demonstrated similar antiproliferative activity. This is noteworthy because it suggests that the identified compounds are likely the main contributors of the extracts’ effects [32]. Both studies identified the extracts’ compounds but failed to apply a mechanistic approach to the results and lacked the use of animal models.

Recently, specialized drug delivery systems such as nanoparticles incorporating OLE have been developed to enhance the antitumor action of olive leaf compounds [94,95].

OOEs also showed significant antiproliferative effects in melanoma and skin cancer models. OC and oleacein inhibited cell migration and induced apoptosis in A431 cells through the suppression of the B-Raf-ERK pathway, although OC was the strongest compound [34]. Fogli et al. (2016) also demonstrated OC’s apoptotic effect in melanoma cells by inhibiting ERK1/2 [77].

Oral administration of an OLE and its main compound (OL) was able to protect against UVB-induced skin damage in vivo with epidermis and extracellular matrix preservation as confirmed histologically. They also prevented increases in MMP, VEGF, COX-2, Ki-67, and CD31 [35]. Additionally, an OOE exhibited stronger antimelanoma activity compared to OC and oleacein. Remarkably, post-treatment with EVOO’s extracts enhanced the toxic effect of higher doses of dacarbazine in A375 but not in HaCaT cells, demonstrating selective cytotoxicity [36]. This study is important because it validates the hypothesis that the entire extract’s action will be stronger than the use of the individual compounds.

According to the literature in Table 2, HT evidenced complex dose-dependent effects under hypoxia in breast cancer cells [43,46]. However, precise dose optimization remains a critical consideration, as excessive concentrations upregulate VEGF expression, promoting angiogenesis [46]. In colorectal cancer models, HT showed anti-inflammatory properties by inhibiting the expression of PGE2, COX-2, and VEGF. Moreover, its impact on HIF-1α demonstrated HT’s potential to disrupt the hypoxic tumor microenvironment [48]. HT’s capacity to modulate tumor progression via interaction with HIF-1α was also reported in breast cancer cells [46]. Additionally, HT induced a cell cycle block at the G2/M phase, mediated through the inhibition of ERK1/2 phosphorylation and downregulation of cyclin D1 expression [47].

HT has shown selective cytotoxicity against prostate cancer cells compared to normal prostate cells by targeting the androgen receptor, which is essential for prostate cancer growth and progression, being a strength of this study. HT also inhibited Akt, STAT3, and NF-κB [53]. However, prostate cancer cells exhibit higher resistance to HT’s antiproliferative effects than breast and colorectal cancer cells, which has been linked to lower H_2_O_2_ accumulation. In addition, HT dose- and time-dependently reduced H_2_O_2_-induced DNA damage in peripheral blood mononuclear cells. A noteworthy aspect of this work is that, at high doses, HT showed pro-oxidant activity and increased DNA damage [52]. This reinforces the need for dose optimization.

Regarding secoiridoids (Table 3), the combination of OL and 2-methoxyestradiol was stronger than individual treatments in inhibiting cell proliferation and migration in osteosarcoma cells, using autophagy induction as a mechanism [85]. Other studies confirmed that the probable mechanistic action of HT and OL in breast cancer cells was through induction of autophagy, although these cells were more sensitive to HT than OL [96,97]. OL aglycone also induced autophagy in neuroblastoma cells through the Ca^2+^-CaMKKβ-AMPK-mTOR pathway, as confirmed in cell and mice models [84]. Similarly, OC decreased viability and inhibited migration of breast cancer cells by inducing Ca^2+^ entry through TRPC6 channels [63]. OC effectively binds and inhibits mTOR phosphorylation, showing potent antiproliferative effects against breast cancer cells, but comparatively it demonstrated poorer activity against colorectal and cervical cancer cells [69]. Another study demonstrated that OL has antiproliferative and cytostatic effects on breast cancer cells, partially mediated by inhibition of PTP1B enzymatic activity, depending on dosage, as shown by molecular docking [60]. OC was shown to bind directly to the ERα ligand-binding domain, having reduced ERα levels in BT-474 cells in silico [62].

Additionally, HT reduced the self-renewal capacity of breast cancer stem cells, inhibited the Wnt/β-catenin pathway, and suppressed EMT by modulating transcription factors like SLUG, SNAIL, and ZEB1. HT’s ability to suppress EMT underlines its potential to reduce migration, invasion, and metastatic potential, particularly in TNBC cells [44]. Similarly, OC reduced tumor growth recurrence, stabilized E-cadherin, decreased vimentin levels, and suppressed HER2 and c-MET receptor activation, potentially via EMT inhibition in mice [65]. Moreover, OC inhibited hepatocellular carcinoma growth and spread by reducing EMT markers and suppressing MMP-2, cyclin D1, and Bcl-2 genes [75].

Notably, a number of studies reported the significant in vivo antitumor efficacy of OC by preventing tumor formation and/or decreasing tumor growth in female nude mice with cancer cell xenografts. Importantly, OC did not cause systemic toxicity in the mice in none of the studies, which is an upside [61,62,63,64,65,66,67]. OC also plays a role in suppressing EMT and inhibiting the activation of important targets in breast cancer therapy such as EGFR, HER2, and c-MET [61,62,63,64,65,66]. OC suppressed HGF-induced proliferation and migration in MDA-MB-231 cells by potentially inhibiting the Brk/paxillin/Rac1 pathway and induced apoptosis via caspases activation [61]. In fact, OC’s potential as a c-MET kinase inhibitor was confirmed in breast and prostate cancer cells. OC also downregulated CD31, a cell adhesion molecule, in endothelial cells [68]. Molecular docking also confirmed the potential of TYR sinapate as a c-MET kinase phosphorylation inhibitor in MDA-MB-231 cells [98]. Additionally, HT and TYR have shown synergic effects in promoting EGFR degradation in vivo, probably through the proteasomal pathway [49].

Wei et al. (2019) demonstrated that MA exerts anticancer effects in colorectal cancer cells by inhibiting cell migration, regulating EMT markers, and activating AMPK while inhibiting the mTOR pathway. Additionally, MA reduced colon carcinogenesis in AOM/DSS mice by attenuating inflammation, tumor growth, and cytokine dysregulation. These findings underscore MA’s potential as a chemopreventive agent for colorectal cancer, primarily through modulation of the AMPK-mTOR pathway [90]. The modulation of this pathway was also verified for OL aglycone in neuroblastoma models [84] and OOEs rich in OC, ligstroside aglycone, elenolic acid, and OL aglycone in liver cancer cells [39].

Both OL and OC demonstrated antiproliferative and apoptotic activities by altering gene expression profiles associated with TNBC. However, OC had a slightly stronger effect than OL [57]. Interestingly, OL was also able to downregulate microRNA expression, exerting antimigratory, apoptotic, and cytotoxic effects in a dose-dependent manner [58].

In colorectal cancer, EVOO’s phenolics caused the upregulation of CB1 (cannabinoid receptor) by reducing DNA methylation at the CNR1 promotor, leading to decreased proliferation in Caco-2 cells. Interestingly, this was confirmed by EVOO supplementation in vivo [33].

In another study, a hydroethanolic OPE extracted using ohmic heating-assisted extraction was the most effective at reducing Caco-2 colorectal cancer cell viability. The study suggests that OPE induces cell death via the mitochondrial pathway with p53 and caspase-3 activation [4]. Another OPE exhibited different activities against colorectal cancer cells. It was cytotoxic, decreased proliferation, and increased OS in HT-29 cells, while it decreased proliferation and cell growth but upregulated VEGF in Caco-2 cells, evidencing the complex response of different cancer cell lines even within the same type of cancer [9]. Different extracts obtained from various olive matrices also showed dose-dependent cytotoxicity against HCT-116 colorectal cancer cells [7].

In leukemia models, an OPE, HT, and verbascoside provided DNA protection against H_2_O_2_-induced genotoxicity while inducing apoptosis via caspase-3 activation [38]. Similar results were observed with an OLE, which inhibited DNA damage, reduced mutation frequencies, and demonstrated dose-dependent cytotoxicity in HL60 cells. Notably, LUT had the highest apoptotic effect [37]. Both studies distinguish themselves from the others by having used the somatic mutation and recombination test with *Drosophila melanogaster*.

The relationship between ROS and cancer is complex. Cancer cells, being highly metabolically active, produce increased ROS levels, which can damage DNA, causing OS which promotes cancer angiogenesis, invasiveness, and metastasis. Low ROS levels regulate hypoxia adaptation and pro-inflammatory cytokine production, while high levels can induce apoptosis and autophagy [21].

In fact, HT showed apoptotic effects by activating the caspase cascade and OS-modulating activities since it increased the activity of catalase, superoxide dismutase, and glutathione peroxidase and reduced thiobarbituric acid reactive substances in colorectal cancer cells [51]. Sun et al. (2014) emphasized the role of ROS generation in HT’s cytotoxic effects. It also highlighted the modulation of the Akt/FOXO3a pathway and the differential expression of antioxidant defense enzymes: superoxide dismutase enhanced HT’s effects while catalase reduced them [22].

In another study, erythrodiol induced apoptosis through ROS production and DNA damage, while oleanolic acid and uvaol induced it by cell cycle arrest. The triterpenes, except for high-dose erythrodiol, protected cells from H_2_O_2_-induced oxidative and DNA damage. The findings highlighted dose-dependent behaviors and emphasized the need for further research on mechanisms and dose optimization [26]. Moreover, OL increased ROS levels in breast cancer cells, showing apoptotic activity [23].

Increased ROS production, higher malondialdehyde and protein carbonyls levels, and decreased glutathione and catalase levels were also observed in colorectal and prostate cancer cells treated with an OLE, composed mostly of chlorogenic acid, showing antimigratory and apoptotic effects. However, all effects were less noticeable in the PC3 cells [27]. Ferreira et al. (2024) also found an increase in malondialdehyde levels in HT-29 colorectal cancer cells treated with an OPE, showing effects on lipid peroxidation [9]. However, all of these studies lack the use of animal models to validate the in vitro results.

In studies with olive secoiridoids, OL alleviated clinical symptoms and reduced colon inflammation, dysplastic lesions, and tumor formation in a mouse model of AOM/DSS-induced colorectal cancer [71]. Similarly, OL-enriched diets prevented preneoplastic lesions across all colon segments, reduced tumor development, and minimized AOM-induced DNA damage in leukocytes [72]. Both studies complement each other effectively, highlighting the protective effects of OL against colorectal cancer. Interestingly, OL also showed a protective effect in mice against cisplatin-induced kidney injury by suppressing OS, inflammation, and apoptosis [86]. In addition, p-HPEA-EDA inhibited tumorigenicity in a chicken embryo chorioallantoic membrane assay, possibly through AMPK activation [73].

Concerning triterpenes, MA did not affect breast cancer and lymphoma cells [26] but interfered with DNA integrity and triggered the apoptotic cascade in colorectal cancer cells [20]. Similarly, erythrodiol showed antiproliferative and apoptotic activities depending on dosage [91]. In addition, MA treatment altered β-actin, moesin and villin 1 expression, indicating cytoskeleton reorganization, which may contribute to its effects on cell cycle arrest, motility, and metastatic potential [88]. MA also inhibited intestinal polyp formation in mice. MA downregulated key components of the Wnt/β-catenin pathway [89]. OL also blocked the Wnt/β-catenin pathway in mice with colorectal cancer [71].

Additionally, MA suppressed the p38 MAPK pathway, causing apoptosis in bladder cancer cells, as evidenced by chromatin condensation and nuclear fragmentation [92]. MA decreased capillary tube formation and VEGF in HUVECs in renal cell carcinoma; this anti-angiogenic effect is significant because this is a highly vascularized tumor [93].

Since most studies focused on apoptotic routes, there is a lack of studies focusing on the anti-invasive, anti-angiogenic, and anti-inflammatory activities of MA and other triterpenes.

HT and OL inhibited cell proliferation and induced apoptosis, likely through the modulation of HIF-1α and p53 expression in colorectal cancer cells. Additionally, HT increased peroxisome proliferator-activated receptor gamma (PPARγ) expression, which may contribute to its tumor-reducing effects [99].

Hazas et al. (2017) investigated the effects of HT and its colonic metabolites, having found that HT and catechol were the only compounds which were extensively metabolized by colorectal cancer cells [100]. In another work, the dose-dependent antiproliferative effects of HT and its lipophilic fractions on a colorectal cancer cell line were dependent on the binding of these compounds to ER-β. Interestingly, HT oleate exhibited a greater antiproliferative effect in comparison to the others due to the higher lipophilicity, enabling easier passage through the cell membrane [101].

In liver cancer, HT inhibited critical oncogenic pathways, including Akt and NF-κB [54]. Zhao et al. (2014) further validated HT’s in vivo efficacy, showing reduced angiogenesis, increased apoptosis, and decreased tumor volume in orthotopic hepatocellular carcinoma models by inhibiting Akt and NF-κB [55]. MMPs are critical in liver cancer angiogenesis, playing roles in extracellular matrix remodeling and affecting tumor progression [102]. OL combined with cisplatin enhanced the inhibitory effects on MMP-7 in HepG2 cells, reducing invasion and metastasis, while increasing caspase-3 expression [74].

Regarding hepatocellular and colorectal cancer, OL inhibited cell growth and induced apoptosis through PI3K/Akt pathway suppression and ROS generation, marked by caspase cleavage, PARP fragmentation, DNA damage, and mitochondrial membrane depolarization [24]. Similar results were obtained in another study in which OC outperformed ibuprofen in inhibiting cell proliferation [25].

In lung cancer, OL induced cell cycle arrest in G2/M phases and apoptosis via the mitochondrial pathway (cytochrome c release) and activated the p38 MAPK pathway in H1299 cells [79]; also, OC inhibited c-MET activation and COX-2 activity, suppressed lung cancer progression, and prevented metastasis in vivo, demonstrating stronger effects in the presence of HGF [80]. Gu et al. (2017) also showed inhibitory effects on lung metastasis in a mice model treated with OC, having decreased Mcl-1, MMP, VEGF and Ki-67 [78].

Other olive secoiridoids also presented interesting bioactivities against neuroblastoma cells: OL induced apoptosis and cell cycle arrest and inhibited migration of SH-SY5Y cells [81]. Oleacein also reduced proliferation through cell cycle arrest, induced apoptosis via Bax and p53 upregulation, Bcl-2 downregulation, and STAT3 inhibition, and impaired cell adhesion and migration in SH-SY5Y cells [83]. OC also exhibited apoptotic and cytotoxic effects, selectively targeting cancer cells by increasing OS markers and inducing apoptosis more effectively in NB2a than BMDN cells [82].

In thyroid cancer cells, OL and Ac-OL inhibited the phosphorylation of ERK and Akt proteins, showing potential as therapeutic agents for thyroid cancers with genetic alterations in the MAPK and Akt pathways. Ac-OL showed higher efficacy than OL, likely due to increased cellular permeability [19].

OPEs inhibited proliferation across various cancer types, including pancreatic, ovarian, lung, prostate, neuroblastoma, and glioblastoma. Interestingly, at lower doses, OPEs improved normal cell viability, suggesting that they could act as protective agents for pancreatic cells [40]. Similarly, OL showed a protective effect towards normal pancreatic cells while promoting caspase-dependent apoptosis in cancer cells (MIA PaCa-2) [103].

Only one study tested olive oil’s phenolics in duos, having found that oleomissional, although less effective on its own, showed strong synergy with OL aglycone, significantly reducing cell numbers across various cancer cell lines. Oleacein, when combined with OL aglycone or ligstroside aglycone, also demonstrated significant synergistic effects in stomach, pancreatic, and lung cancer cell lines. Additionally, breast cancer and melanoma cells showed high responsiveness to these combinations, with substantial reductions in viability. The study also found that olive oils containing higher quantities of oleocanthal exhibited stronger cytotoxic effects compared to those without [41].

This is in accordance with Goren et al. (2019), who showed that EVOOs with higher OC content were more toxic to cancer cells, whereas EVOOs with low or no OC had little effect on cell viability [70]. Additionally, OC treatment resulted in a rapid loss of breast and prostate cancer cell viability while having minimal effect on normal cells. OC induced necrotic cell death in cancer cells, mainly through lysosomal membrane permeabilization, leading to the release of cathepsins into the cytosol, causing rapid cell death [70].

As shown in Table 5, oleic acid and TYR have the potential to inhibit inflammatory responses and angiogenic activities in glioblastoma cells by inhibiting COX-2, ERK and JNK and decreasing PGE2. Both compounds were also able to inhibit endothelial cells’ migration [104]. Indeed, olive matrices are a great source of oleic acid and dietary consumption of this fatty acid has been linked with a reduced incidence of pancreatic cancer due to a reduction in hyperinsulinaemia and consequent DNA mutations which can induce tumor growth [105].

## 4. Conclusions

To sum up, the olive compounds that appear to be the most effective against cancer are those extensively studied in both in vitro and in vivo studies, namely HT, OL, OC, and MA.

In terms of phenolic alcohols, HT presented antiproliferative, apoptotic, anti-angiogenic, antimigratory, anti-invasive, and antimetastatic effects, especially on breast and colorectal cancer, by modulating key pathways and molecules involved in cancer progression, such as EMT, EGFR, PI3K/Akt/mTOR, NF-κB, STAT3, ERK1/2, HIF-1α, PGE2, COX-2, VEGF, ROS, PARP1, FAS, and PPARγ.

Regarding secoiridoids, OL exhibited antiproliferative, apoptotic, antimigratory, and anti-invasive effects, predominantly on breast and colorectal cancer, being able to modulate gene expression, COX-2, NF-κB, ROS, and PTP1B. OC demonstrated antiproliferative, apoptotic, anti-angiogenic, antimigratory, anti-invasive, and antimetastatic effects, mainly on breast cancer, by targeting ERα, HER2, EGFR, and c-MET.

Concerning triterpenes, MA showed antiproliferative, apoptotic, anti-angiogenic, and antimigratory effects, especially on colorectal cancer, mostly through the apoptotic intrinsic pathway.

All four compounds showed selective cytotoxicity against cancer cells. Additionally, HT, OL, and OC showed synergistic effects when combined with anticancer drugs, being able to improve their efficacy. Furthermore, HT, OL and MA showed protective effects against several side effects of chemotherapy.

Most of these articles emphasize the importance of compound/extract dose optimization, because very low doses are sometimes ineffective, whereas high doses can counteract the beneficial effects, for example, being pro-oxidant.

Overall, these findings underscore the potential of olive compounds as therapeutic agents in nutraceuticals, functional foods, and anticancer drug formulations. However, further research is needed to clarify the exact mechanism of action of each compound, since the articles target different signaling pathways and molecules, hindering cross-comparison between studies.

To the best of our knowledge, there are still no studies using OPEs with mice models, which seems to be an opportunity for future research.

## Figures and Tables

**Table 1:** Antitumor activity of olive extracts.

Comp.	Cancer	Effects	Study	Model	Dose	Ref.
Elenolic acid, HT, OL aglycone, TYR	Breast	Antiproliferative, apoptotic, OL aglycone synergy w/trastuzumab	In vitro	MCF-7, SKBR3, SKBR3/Tzb100	6.25–100 μM, 1–5 d	[28]
Elenolic acid, HT, OL derivatives, TYR, etc.		Antiproliferative, apoptotic, cytotoxic		MCF-7, SKBR3	25–100 μM, 6 h–4 d	[29]
		FAS inhibition			50 μM, 48 h	[30]
HT, HT glucoside, OL, OL derivatives, VER		Antiproliferative, HT synergy w/5-fluorouracil		MDA-MB-231	Extracts (0.1–20 μg/mL), HT (162–2595 μM), 48 h	[5]
OL, OL derivatives, oleanolic acid, etc.		Cytotoxic		JIMT-1, MCF-7, SKBR3	10–1000 μg/mL, 72 h	[31]
HT, HT acetate, LUT, LUT derivatives, OL	Breast, bladder, endothelial	Antiproliferative		BBCE, MCF-7, T-24	0–50 μM, 2 d	[32]
EVOO’s compounds, HT	Colorectal	Antiproliferative	In vitro, in vivo	Caco-2, NCM460; Sprague Dawley rats	50 μM, 24–48 h; 250 μL/300 g of EVOO: single intake at day 10 or daily for 10 d	[33]
Apigenin, caffeic acid, HT, OL, quercetin, TYR, etc.		Antiproliferative, apoptotic, selective cytotoxicity, protective	In vitro	Caco-2	1.5 mg/mL, 72 h	[4]
Erythrodiol, HT, MA, OC, OL aglycone, oleacein, etc.		Cytotoxic		HCT-116	88.25–875.5 μg/mL, 72 h	[7]
Gallic acid, catechin, caffeic acid, kaempferol, etc.	Colorectal, prostate	Cytotoxic, antimigratory, apoptotic		HT-29, PC3	198.6–535.3 μg/mL, 12–72 h	[27]
OOE’s compounds, HT, OC, oleacein, TYR	Skin	Antiproliferative, antimigratory, apoptotic		A431, HaCat	1–200 μM, 72–144 h	[34]
OLE’s compounds, OL		Anticarcinogenic, anti-inflammatory	In vivo	Albino hairless mice	10–1000 mg/kg, 2x/d, 30 wk, oral	[35]
EVOO’s compounds, OC, oleacein	Melanoma	Antiproliferative, selective cytotoxicity	In vitro	A375, A375M, HaCaT	0.025–0.4% (*v*/*v*), 24–48 h	[36]
OLE’s compounds, OL, LUT	Leukemia	Antigenotoxic, antimutagenic, apoptotic, cytotoxic, protective	In vitro, in vivo	HL60; *D. melanogaster*	3.75–160 μL/mL, 2–640 μM, 72 h	[37]
OPE’s compounds, HT, TYR, VER		Antigenotoxic, antimutagenic, apoptotic, antiproliferative, protective			3.75–320 μL/mL, 6.25–480 μM, 72 h	[38]
Elenolic acid, ligstroside aglycone, OC, OL aglycone	Liver	Antiproliferative, apoptotic, cytotoxic, autophagy activation	In vitro	Hep3B, HepG2, Huh7	4.81–9.62 μg/mL, 24–72 h	[39]
OPE’s compounds	Breast, colon, glioblastoma, lung, neuroblastoma, ovarian, pancreatic, prostate, skin	Antiproliferative, selective cytotoxicity		MIA PaCa-2, HT-29, A2780, H460, A431, Du145, BE2-C, MCF-7, U-87, SJ-G2, SMA, MCF-10A, HPDE	0–200 μg/mL, 72 h	[40]
OC, oleacein, ligstroside aglycone, OL aglycone, oleomissional	Breast, cervix, colon, gastric, liver, lung, melanoma, pancreas	Antiproliferative, selective cytotoxicity		MDA-MB-231, SK-BR-3, MCF-7, SK-MEL-28, A2058, HT-29, AGS, HepG2, PANC-1, H1299, Hela, HaCaT, MCF-10A	Half of the EC_50_, 72 h	[41]
Comselogoside, elenolic acid, HT, LUT, MA, VER, etc.	Breast, pancreatic, colorectal	Antiproliferative, cytotoxic, anti-angiogenic		MCF-7, AsPC-1, HT-29, Caco-2	100 mg/mL, 24 h	[9]

EC_50_ (half maximal effective concentration), EVOO (extra virgin olive oil), FAS (fatty acid synthase), HT (hydroxytyrosol), LUT (luteolin), MA (maslinic acid), OC (oleocanthal), OL (oleuropein), OLE (olive leaf extract), OOE (olive oil extract), OPE (olive pomace extract), TYR (tyrosol), VER (verbascoside).

**Table 2:** Antitumor activity of olive phenolic alcohols.

Comp.	Cancer	Effects	Study	Model	Dose	Ref.
HT	Breast	Antiproliferative, cytotoxic	In vitro	MCF-7	5–600 μM, 16 h	[43]
		Anti-invasive, antimigratory, antiproliferative, antimetastatic		BT549, Hs578T, MDA-MB-231, SBE-HEK293, SUM159PT	0.5–100 μM, 72 h	[44]
		Antiproliferative, antitumorigenic, protective, synergy w/paclitaxel	In vitro, in vivo	MCF-7, MDA-MB-231; Sprague Dawley rats	10–100 μM, 72 h; 0.5–2 mg/kg/d, 6 wk, oral	[45]
		Antitumorigenic	In vitro, in silico	MCF-7	5–400 μM, 16 h	[46]
	Colorectal	Antiproliferative	In vitro	Caco-2	HT (5–162.5 µM, 15 m-96 h)	[47]
		Anti-angiogenic, anti-inflammatory, antitumorigenic	In vitro, in vivo	HT-29, WiDr; mice w/HT-29 xenograft	50–100 μM, 3–48 h; 10 mg/kg/d, 14 d	[48]
		Apoptotic, selective cytotoxicity	In vitro	CRL1807, DLD1	50–200 μM, 24, 48 h	[22]
HT, TYR		Selective cytotoxicity, antiproliferative, antitumorigenic	In vitro, in vivo	Caco-2, CCD-18Co, HT-29, WiDr; mice w/HT-29 xenograft	1–300 μM, 2–48 h; 10 mg/kg/d, 14 d, intraperitoneal	[49]
HT		Antiproliferative, selective cytotoxicity, synergy w/cetuximab, activated autophagy	In vitro	Caco-2, CCD-18Co, HaCaT, HT-29, WiDr	1–300 μM, 8–48 h	[50]
		Apoptotic		LS180	50–150 μM, 24 h	[51]
	Breast, colorectal, prostate	Antiproliferative, protective		HCT, LNCAP, MCF-7, MDA, PBMC, PC3, SW480	10–100 μM, 0.5–24 h	[52]
	Prostate	Antiproliferative, apoptotic, selective cytotoxicity		C4-2, LNCaP, RWPE1, RWPE2	10–400 μM, 24–72 h	[53]
	Liver	Anti-inflammatory, antiproliferative		Hep3B, HepG2	30–200 μM, 48–72 h	[54]
		Anti-angiogenic, antiproliferative, apoptotic, selective cytotoxicity, antitumorigenic	In vitro, in vivo	Hep3B, HepG2, HL-7702, Huh-7, SK-HEP-1; HCC mice	100–400 μM, 48–72 h; 10–20 mg/kg/d, 3 wk, intraperitoneal	[55]

HCC (hepatocellular carcinoma), HT (hydroxytyrosol), TYR (tyrosol).

**Table 3:** Antitumor activity of olive secoiridoids.

Comp.	Cancer	Effects	Study	Model	Dose	Ref.
OC, OL	Breast	Antiproliferative, apoptotic	In vitro	MDA-MB-231, MDA-MB-468	OC: 250 μM, OL: 500 μM, 12–48 h	[57]
OL		Antimigratory, antiproliferative, apoptotic, cytotoxic		MCF-7	150–2400 μg/mL, 24–72 h	[58]
		Antiproliferative, apoptotic, selective cytotoxicity, antitumorigenic, synergy w/doxorubicin	In vitro, in vivo	MCF-10A, MDA-MB-231; mice injected w/MDA-MB-231	50 mg/kg, 72 h; 50 mg/kg, 4 wk, 1X/wk, intraperitoneal	[59]
		Anti-invasive, antimigratory, apoptotic	In vitro	MCF-7, MDA-MB-231	12.5–100 μM, 24–72 h	[23]
		Antiproliferative, cytostatic	In vitro, in silico	MCF-7	0.98–250 μM, 24–48 h	[60]
OC		Anti-invasive, antimigratory, antiproliferative, apoptotic, selective cytotoxicity	In vitro, in vivo	BT-474, MCF-7, MCF-10A, MDA-MB-231, mice w/MDA-MB-231 xenograft	5–15 μM, 24–72 h; 5 mg/kg, 3X/wk, intraperitoneal	[61]
		Antiproliferative, antitumorigenic, selective toxicity, synergy w/tamoxifen	In vitro, in vivo, in silico	BT-474, MCF-7, T-47D, mice w/BT-474 xenograft	5–60 μM, 24–48 h; 5–10 mg/kg, 3X/wk, 43 d, intraperitoneal	[62]
		Antimigratory, antiproliferative, selective cytotoxicity	In vitro	MCF-7, MCF-10A, MDA-MB-231	10–20 μM, 24–72 h	[63]
		Anti-invasive, antimigratory, antiproliferative, selective cytotoxicity, antitumorigenic, synergy w/lapatinib	In vitro, in vivo	BT-474, MCF-12A, SKBR3, mice w/BT-474 xenograft	5–80 μM, 24–48 h, 10 mg/kg, 3X/wk, intraperitoneal	[64]
		Antitumorigenic, selective toxicity	In vivo	Mice w/BT-474 or MDA-MB-231 xenograft	10 mg/kg/d, 40 d, oral	[65]
			In vivo, in silico	Mice w/MDA-MB-231 xenograft	10 mg/kg/d, 15–70 d, oral	[66]
		Antiproliferative, selective cytotoxicity, antitumorigenic	In vitro, in vivo	MDA-MB-231, MDA-MB-468, BT-474, MCF-7, MCF10A; Foxn1^nu^/Foxn^1+^ mice	2.5–80 μM; 10 mg/kg, 7X/wk, 40 d, oral	[67]
OC	Breast, prostate	Anti-angiogenic, anti-invasive, antimigratory, antiproliferative	In vitro, in silico	MCF-7, MDA-MB-231, PC-3	2–100 μM, 24 h	[68]
	Breast, cervical, colorectal	Antiproliferative		Caco-2, HeLa, MCF-7, MDA-MB-231, T47D	0.1–50 μM, 24–72 h	[69]
	Breast, pancreas, prostate	Antiproliferative, selective cytotoxicity, antitumorigenic	In vitro, in vivo	BJ-hTert, MCF-7, MCF-10A, MDA-MB-231, HEK-293T, PC3, PNET mice	1–100 μM, 1–24 h; 5 mg/kg/d, 5 wk, intraperitoneal	[70]
OL	Colorectal	Preventive against colitis	In vivo	C57BL/6 mice	50–100 mg/kg, 63 d, oral	[71]
		Preventive against carcinogen (azoxymethane)		A/J mice w/azoxymethane-induced tumors	125 mg/kg, 6 wk, 1X/wk, intraperitoneal	[72]
p-HPEA-EDA		Apoptotic, antitumorigenic, inhibited colony formation	In vitro, in vivo	HCT-116, HT-29, JB6Cl41, SKBR3; chicken embryo chorioallantoic membrane	0.1–10 μg/mL, 12–48 h; 50 μg/mL, 3 d	[73]
OL	Colorectal, liver	Antiproliferative, apoptotic	In vitro	HepG2, Huh7, RKO	10–80 μM, 24 h	[24]
OC		Antiproliferative, apoptotic, selective cytotoxicity		Hep3B, HepG2, HT-29, Huh7, PLC/PRF/5, SW480	0.78–100 μM, 24–72 h	[25]
OL	Liver	Synergy w/cisplatin, cytotoxic		HepG2	100–400 μM, 24–48 h	[74]
OC		Anti-invasive, antimigratory, antiproliferative, apoptotic, selective cytotoxicity, antitumorigenic, antimetastatic	In vitro, in vivo	HCCLM3, HepG2, Huh-7, LO2; HCC mice	10–80 μM, 24–72 h; 5–10 mg/kg/d, 5 wk, intraperitoneal	[75]
OL	Melanoma	Antiproliferative, apoptotic, cytotoxic, synergy w/dacarbazine and everolimus	In vitro	A375, M21, WM266–4	250–800 μM, 24–72 h	[76]
OC		Antiproliferative, apoptotic, selective cytotoxicity		A375, 501Mel, HDFa	0.01–50 μM, 72 h	[77]
OC	Melanoma, lung	Anti-angiogenic, anti-invasive, antimigratory, antiproliferative, apoptotic, antitumorigenic, antimetastatic	In vitro, in vivo	A2058, A375, HaCaT, HUVEC; mice w/A375 xenograft; lung metastasis model	5–60 μM, 24–48 h; 10 mg/kg/d, 3 wk, intraperitoneal; 15 mg/kg/d, 6 wk	[78]
OL	Lung	Antiproliferative, apoptotic	In vitro	H1299	50–200 μM, 24 h	[79]
OC		Antimigratory, antiproliferative, selective cytotoxicity, antitumorigenic, antimetastatic	In vitro, in vivo	A549, HMVEC, NCI-H322M; Foxn1^nu^/Foxn^1+^ mice injected w/A549	1–60 μM, 24–72 h; 10 mg/kg/d, 8 wk, oral	[80]
OL	Neuroblastoma	Anti-invasive, antimigratory, antiproliferative, apoptotic	In vitro	SH-SY5Y	25–800 μM, 24–72 h	[81]
OC		Apoptotic, selective cytotoxicity		BMDN, NB2a	0.1–1000 μM, 24 h	[82]
Oleacein		Antimigratory, antiproliferative, apoptotic, selective cytotoxicity		SH-SY5Y, WI-38	1–50 μM, 24–72 h	[83]
OL aglycone		Autophagy induction	In vitro, in vivo	SH-SY5Y; TgCRND8 mice	50 μM, 5 m-5 h; 50 mg/kg of diet, 8 wk, oral	[84]
OL	Osteosarcoma	Antimigratory, antiproliferative, autophagy induction, synergy w/2-methoxyestradiol	In vitro	143B	1–250 μM, 24–60 h	[85]
OL	-	Protective effect against acute renal injury caused by cisplatin	In vivo	BALB/cN mice	20 mg/kg, oral gavage	[86]
OL, Ac-OL	Thyroid	Antiproliferative, selective cytotoxicity	In vitro	BCPAP, TAD-2, TPC-1	10–100 μM, 24–48 h	[19]

Ac-OL (peracetylated derivative of oleuropein), HCC (hepatocellular carcinoma), HUVEC (human umbilical vein endothelial cells), OC (oleocanthal), OL (oleuropein), p-HPEA-EDA (dialdehydic form of decarboxymethyl ligstroside aglycone), PNET (pancreatic neuroendocrine tumor).

**Table 4:** Antitumor activity of olive pentacyclic triterpenes.

Comp.	Cancer	Effects	Study	Model	Dose	Ref.
MA	Colorectal	Apoptotic, clastogenic, cytotoxic	In vitro	HT-29	61 μM, 12–72 h	[20]
		Antiproliferative, cytotoxic			3.75–30 μM, 3–72 h	[88]
		Antitumorigenic, chemopreventive, protective, apoptotic	In vivo	Apc^Min/+^ mice	100 mg/kg of feed, 6 wk, oral	[89]
		Antimigratory, antiproliferative, apoptotic	In vitro, in vivo	HCT116, SW480; AOM/DSS mice; BALB/c mice w/HCT116 xenograft	5–20 μM, 12–36 h; 10–30 mg/kg every 2 d, 17 d, oral gavage	[90]
Erythrodiol		Antiproliferative, apoptotic	In vitro	HT-29	10–150 μM, 24–72 h	[91]
MA	Bladder	Apoptotic, selective cytotoxicity, antitumorigenic	In vitro, in vivo	253J, L-02, MRC-5, RT4, T24, TCCSUP, PBC-1, PBC-2; BALB/c mice xenograft model	1–1000 μM, 48–96 h; 5–20 mg/kg every other day, intraperitoneal	[92]
	Kidney	Anti-angiogenic, antiproliferative, selective cytotoxicity	In vitro	ACHN, Caki-1, HUVEC, PTEC, SN12K1	47.11–97.04 μM, 24 h	[93]
Erythrodiol, MA, oleanolic acid, uvaol	Breast, lymphoma	Antiproliferative, apoptotic, cytotoxic		MCF-7, MDA-MB-231, U937	12.5–100 μM, 24–120 h	[26]

AOM/DSS (azoxymethane/dextran sulfate sodium), HUVEC (human umbilical vein endothelial cells), MA (maslinic acid).

**Table 5:** Antitumor activity of olive compounds from different classes.

Comp.	Cancer	Effects	Study	Model	Dose	Ref.
OC, ligstroside aglycone, TYR derivatives	Breast	Anti-invasive, antimigratory, antiproliferative, selective cytotoxicity	In vitro, in silico	MDA-MB-231, MCF-10A	10–100 μM, 24–48 h	[98]
HT, OL		Anti-invasive, antimigratory, antiproliferative	In vitro	MDA-MB-231	0–150 μM, 24–48 h	[96]
				MCF-7, T47D		[97]
	Colorectal	Antiproliferative, apoptotic		HT-29	200–800 μM, 24–72 h	[99]
HT, HT butanoate, octanoate and oleate		Antiproliferative		HCT8-β8n	5–50 μM, 20–80 h	[101]
HT, catechol, PA, PP, HPP, DHPP		Antiproliferative, apoptotic, cytotoxic		Caco-2, HT-29	100–200 μM, 8–48 h	[100]
HT, TYR, OL, oleic acid	Glioblastoma	Anti-angiogenic, anti-inflammatory, antimigratory		HBMEC, U-87	100 μM, 24 h	[104]
HT, OL	Pancreatic	Antiproliferative, apoptotic, selective cytotoxicity		ASPC-1, BxPC-3, CFPAC-1, HPDE, MIA PaCa-2	0–300 μM, 24–48 h	[103]

DHPP (dihydroxyphenylpropionic acid), HPP (hydroxyphenylpropionic acid), HT (hydroxytyrosol), OC (oleocanthal), OL (oleuropein), PA (phenylacetic acid), PP (phenylpropionic acid), TYR (tyrosol).

## Data Availability

Data are contained within the article.

## Abbreviations

Ac-OL (peracetylated derivative of oleuropein), Akt (protein kinase B), AOM/DSS (azoxymethane/dextran sulfate sodium), AMPK (adenosine monophosphate-activated protein kinase), AP-1 (activating protein 1), CaMKKβ (Ca^2+^/calmodulin-dependent protein kinase kinase beta), c-MET (mesenchymal–epithelial transition factor), COX (cyclooxygenase), DHPP (dihydroxyphenylpropionic acid), EC_50_ (half maximal effective concentration), EGF (epidermal growth factor), EGFR (epidermal growth factor receptor), EMT (epithelial–mesenchymal transition), ER (estrogen receptor), ERK (extracellular signal-regulated kinase), FAS (fatty acid synthase), GLUT (glucose transporter), HCC (hepatocellular carcinoma), H_2_O_2_ (hydrogen peroxide), HGF (hepatocyte growth factor), HIF (hypoxia inducible factor), HUVECs (human umbilical vein endothelial cells), IL (interleukin), JNK (c-Jun N-terminal kinase), LUT (luteolin), MA (maslinic acid), MAPK (mitogen-activated protein kinase), MMP (matrix metalloproteinase), MMP (matrix metalloproteinase), mTOR (mammalian target of rapamycin), NF-κB (nuclear factor-kappa B), OC (oleocanthal), OL (oleuropein), OLE (olive leaf extract), OOE (olive oil extract), OP (olive pomace), OPE (olive pomace extract), OS (oxidative stress), PARP (poly (ADP-ribose) polymerase), PBMCs (peripheral blood mononuclear cells), PGE2 (prostaglandin E2), PNET (pancreatic neuroendocrine tumor), PI3K (phosphatidylinositol-3-kinase), PPAR (peroxisome proliferator-activated receptor), PP (phenylpropionic acid), RNS (reactive nitrogen species), ROS (reactive oxygen species), STAT (signal transducer and activator of transcription), TGF (transforming growth factor), TNF (tumor necrosis factor), TNBC (triple negative breast cancer), TYR (tyrosol), VEGF (vascular endothelial growth factor).
